# Sensory Analyses Driven Formulation of Fruit Cereal Bars Enriched With *Arthrospira platensis* Dried Powder

**DOI:** 10.1002/fsn3.70154

**Published:** 2025-04-25

**Authors:** Antonio Ottombrino, Marta Cianciabella, Chiara Medoro, Adelia Picariello, Annachiara Oliviero, Vincenzo De Sena, Stefano Predieri, Mauro Rossi

**Affiliations:** ^1^ Institute of BioEconomy, CNR Michele all'Adige‐TN Italy; ^2^ Institute of BioEconomy, CNR Bologna Italy; ^3^ GMF Oliviero F.lli s.r.l., Food Quality Laboratory Monteforte Irpino‐AV Italy; ^4^ Institute of Food Sciences, CNR Avellino Italy

**Keywords:** anti‐oxidant properties, sensory analysis, soft cereal bars, spirulina

## Abstract

Cereal bars represent a fast source of nutrients and energy, instrumental in enlarging the functional foods market. Among the different bioactive components, Arthrospira platensis (spirulina) has gained great interest for application in functional food, thanks to its pigment phycocyanin, a high‐value protein, with antioxidant properties. The bar's nutritional composition was planned to produce enriched fruit cereal bars with an increased protein content of up to 10%, with a 2% spirulina addition. Bars were prepared by mixing sugars, honey, puffed wheat, and dried fruits (candied orange and lemon, peanuts, and almonds) at 130°C. Finally, different concentrations of spirulina powder (0.2%, 1.0%, and 2.0%) were added by mixing with heating off. Sensory evaluation was conducted by a trained panel on the enriched bars at three different spirulina concentrations. Tests included Descriptive Analysis (DA), Check‐All‐That‐Apply questionnaire (CATA), and Temporal Dominance of Sensations (TDS). Sensory analysis indicated that olfactory and flavor notes were essentially red fruits, raisins, and toasted and spicy odor at all spirulina concentrations, without any trace of off‐odors or ‐flavors. In the 0.2% spirulina bar the attribute “citrus” was dominant during the whole taste analysis, while 1% and 2% bars showed the dominance of “red and dried fruit” flavors. Enriched fruit cereal bars with spirulina up to 2% showed increased levels of a high‐value protein, without inducing undesirable changes in sensory parameters. These are encouraging data for successful spirulina‐supplemented functional foods production.

## Graphical abstract

Design of enriched cereal bars with an increased protein content, spirulina addition, and combination of high nutritional values with optimal organoleptic features.

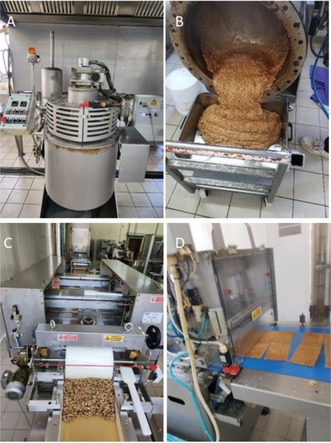

## 1 Introduction

Cereal bars traditionally represent a good meal substitute, as they are a fast source of nutrients and energy. The demand for easy and immediate nutrient‐dense meal replacement has been rising for the past two decades. More recently, cereal bars have been considered instrumental in enlarging the functional food market. A recent consumer study reports that bars were rated as tasty but also unhealthy, highly caloric, and processed, hence judged by adults not suited for children (Saraiva et al. 2024). Kumar et al. (2018) explored spirulina enriched protein bars sensory traits, with an adult panel, to select those more suitable for children, on the base of previous studies, indicating how for children taste liking is a key predictor of food choice (Nguyen et al. 2015). Thus it is important to guarantee both hedonic and nutritional‐functional properties to reach consumers' expectations (Ballco et al. 2020). Dried fruit‐based bars are favorable matrices for functional bars, providing highly nutritious products containing natural sugars, vitamins, minerals, and other bio‐nutritive components (Ayad et al. 2020). Among the different bioactive components, the cyanobacteria *Arthrospira plate*nsis (commonly known as spirulina) has gained great interest for application in functional food products because of its high nutritional, as well as peculiar composition (Gershwin and Belay 2008). *A. platensis* belongs to the phylum cyanobacteria commonly referred to as blue‐green alga with an interesting nutritional composition, especially for its high protein content, γ‐linolenic acid, and phycocyanin contents (Maddiboyina et al. 2023). Especially, the bright blue polypeptide pigment C‐phycocyanin appears to be linked to metallo‐protective, antioxidant, and anti‐inflammatory activities (Liu et al. 2022; Maddiboyina et al. 2023). Spirulina protein content ranges between 60% and 70% dry weight, and C‐phycocyanin is the major protein component (Khan et al. 2005). Furthermore, since these proteins contain all the essential amino acids, they are considered high‐quality proteins (Khan et al. 2005). Carbohydrates and lipids represent 15%–20% and 5%–10%, respectively, of its dry weight. The latter are a good source of essential fatty acids like ‐linolenic and cis‐linoleic acids (Quoc et al. 1994). Importantly, spirulina also retains vitamin B12's high content, generally stored in meat, and beta‐carotene, the precursor of vitamin A, as well as important levels of iron, calcium and phosphorous (Khan et al. 2005). However, there is much variation in the overall percentage of added spirulina powder in functional food. Few studies have investigated the algae introduction, mainly seaweed, in cereal bars (Masturah et al. 2015; Udayangani et al. 2019). However, the main pitfall in these studies was masking the algae flavor and promoting consumer acceptance. Indeed, consumers preferred the gluten‐free fresh pasta standard sample, without spirulina addition, then the enriched one, mainly because of the flavor component (Fradinho et al. 2020). A good strategy to enhance consumer acceptance of high spirulina percentages was to adopt the coaxial printing methodology and hide spirulina inside the food matrix (Uribe‐Wandurraga et al. 2020).

A better solution to overcome these biases related to the novel ingredients beneficial for health would be consumer information through adequate nutrition labeling to help consumers in evaluating the healthfulness of products and foster healthful food choices (Centurión et al. 2019). In particular, front‐of‐pack nutrition labeling (Ares et al. 2018) and, for smarter consumers, also technological labeling are of major importance (Htun et al. 2023). This communicative support would increase customer trust and also counteract food neophobia, which may raise refusal both based on lack of familiarity and the presence of unusual appearances, odors, and flavors (La Barbera et al. 2016).

A suitable tool to respond to consumers' novel product acceptance is sensory analysis. Indeed, it gave the chance to assess product features and improve them during the development phase, before arriving on the market and to consumer judgment. The integrated use of different sensory tests provides a deep knowledge of the product's sensory profile and contributes to predicting consumer appreciation. Descriptive analysis (DA) (Stone et al. 2004) is effective in outlining the relative intensity of sensory attributes, to check if the final product fulfills producer expectations; Check‐All‐That‐Apply (CATA) methodology (Ares et al. 2010) identifies attributes actually perceived by assessors, Temporal Dominance of Sensation (TDS) (Labbe et al. 2009; Schlich 2017) allows for monitoring the dynamics of relevant attributes during tasting, including, if present, unpleasant aftertastes.

The present study aimed to develop an innovative cereal bar on an industrial scale by mixing dried fruits with specifically defined amounts of spirulina powder, creating a functional‐tasty snack. The innovative bars were sensorially analyzed to define their sensory features and assess the presence of off‐odors and/or off‐flavors, potentially impacting consumer appreciation.

## 2 Materials and Methods

### 2.1 Raw Materials

The raw materials were acquired as follows. Sucrose (Italia Zuccheri, Minerbio‐BO, Italy), honey (Apicoltura De Simone, Monte di Procida‐NA, Italy), puffed wheat (Lameri Spa, San Bassano‐CR, Italy), candied orange and lemon cubes (Ambrosio IDAV Spa, Striano‐NA, Italy), toasted peanuts (Murano Spa, Pomigliano d'Arco‐NA, Italy), granulated almonds (Alfrus Srl, Modugno‐BA, Italy), sunflower oils (Basso Fedele Srl; San Michele di Serino‐AV, Italy), lemon and orange essential oils, glycerol, and glucose (Chimpex Industriale Spa, Pascarola‐NA, Italy) were all purchased as food‐grade ingredients. Spirulina was purchased as a food‐grade freeze‐dried powder from Biospira srl (Alvignano‐CE, Italy).

### 2.2 Cereal Bar Formulation and Nutritional Profile

An innovative recipe for cereal bars was formulated (Table 1), defining a specific combination of candied and dried fruits to increase nutritional values, while mitigating the spirulina's organoleptic features. As specified by the European law 1169/2011 on mandatory food labeling, the ingredients' average values to be declared can be obtained from three sources: (I) laboratory analyses; (II) calculations carried out starting from actual average values related to the used ingredients; (III) calculation based on generally established and accepted data. Considering that ingredient distribution within the bars is unavoidably heterogeneous, hampering a statistically significant laboratory analysis of components, this study adopted option III: the innovative cereal bars, enriched with spirulina, were then designed on the basis of nutritional tables from the U.S. Department of Agriculture (U.S. Department of Agriculture FoodData Central, USDA FoodData Central). Moisture was calculated by adopting the Loss on Drying (LoD) method and using a MB90 moisture analyzer (OHAUS Europe GmbH, Nänikon Switzerland). LoD is expressed as a percentage (w/w) resulting from water and any volatile matter that can be driven off under specified conditions.

**TABLE 1:** Bars' composition (g/100 g).^a^

Food component	Protein	Total lipids	Saturated lipids	Total carbohydrates	Soluble sugar	Fibers	kcal	% in bar
Sucrose granulated	0	0	0.36	0	99.6	0	401	6.72
Glucose	0	0	0	0	100.0	0	407	16.81
Honey	0.3	0	0	0	82.4	0.2	304	8.4
Puffed wheat	17.6	0	0	76.5	0	11.8	353	6.05
Candied orange cubes	7.14	0	0	0	60.7	31.0	357	11.80
Candied lemon cubes	7.14	0	0	0	60.7	31.0	357	9.24
Toasted peanuts	24.6	50.3	7.8	21.4	5.0	8.5	596	24.40
Granulated almonds	21.4	51.1	7.8	20	0.2	10.8	626	10.01
Glycerol	0	0	0	0	0	0	275	3.03
Sunflower oil	0	93.2	8.9	0	0	0	884	3.03
Orange essential oil	0.9	0.1	0	12	0	0	47	0.25
Lemon essential oil	1.1	0.3	0	9	0	0	29	0.25
Spirulina powder	57.5	7.7	0	23.9	3.1	3.6	290	0–2.0

^a^ Data derived from USDA FoodData Central (U.S. Department of Agriculture, Agricultural Research Service).

### 2.3 Industrial Manufacture of the Functional Bars

**FIGURE 1:**
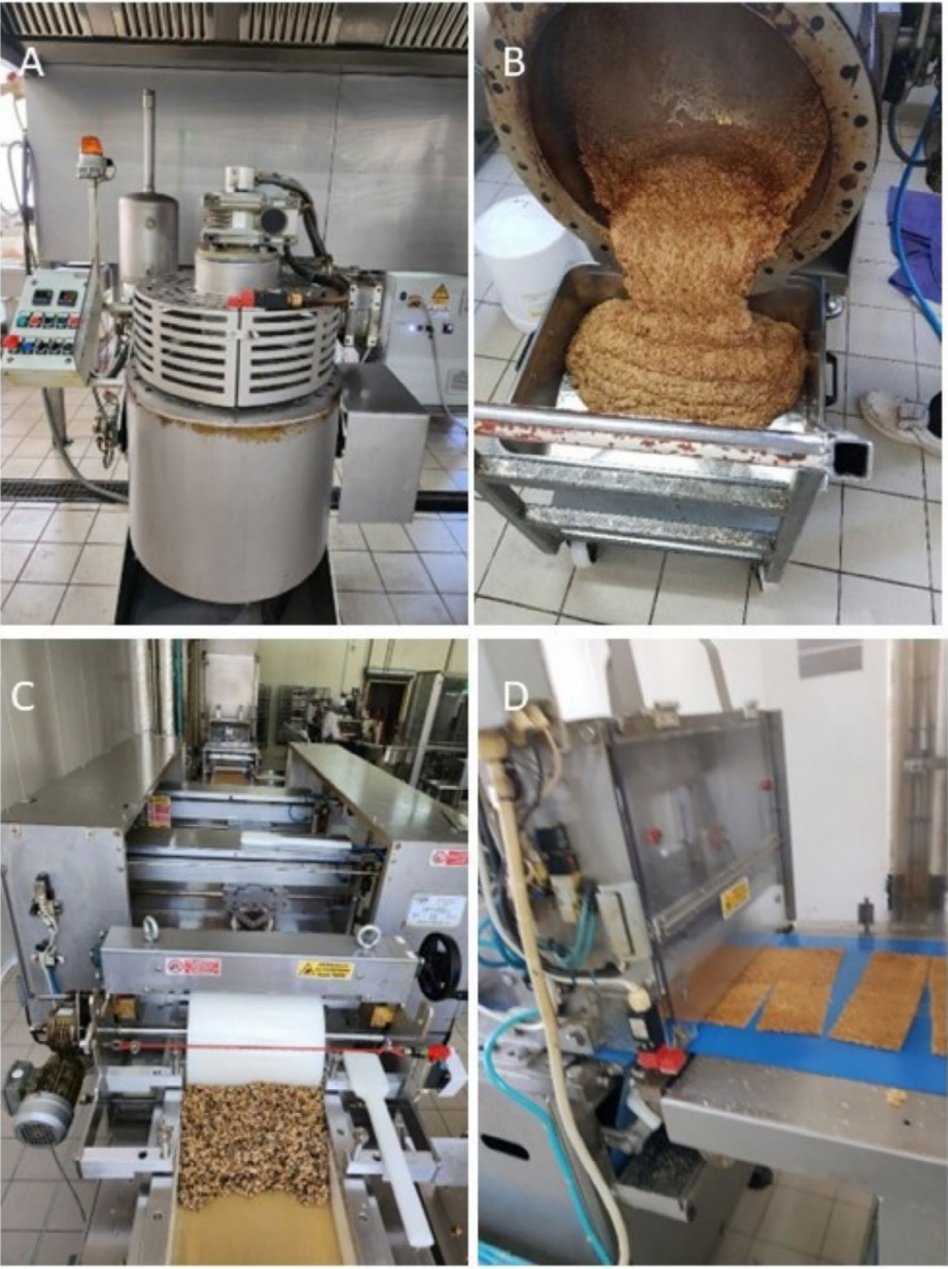
Instrumentation for pilot‐scale production of Spirulina cereal bars. (A) Industrial cooker equipped with a double‐jacketed stainless‐steel vessel and a mixing system; the binders (a mixture of sucrose, glucose, and honey) were poured off into the cooker, followed by the addition of dried components with spirulina powder and continuous stirring. (B) Dough recovery. (C) The dough was molded in a stainless steel tray and (D) cut into bars by an automatic steel bar cutting machine.

The experimental bars were prepared as follows. Sugars and honey were preliminarily mixed for 15 min in a pre‐heated (130°C) C3‐AUTO cooker (MIA Food Tech, Castiglione Falletto‐CN, Italy), equipped with a 110 L double‐jacketed stainless‐steel vessel with oil and electric heating (Figure 1, left). The mixing system allowed for a double planetary movement to mix ingredients, eliminating water and homogenizing the dough. Then, dry ingredients (puffed wheat, candied orange and lemon cubes, toasted peanuts, and granulated almonds) were added in the ratio indicated in Table 1. The mixture was continuously mixed (30 rpm) for 5 min with heating off. Then, the other technological components were added by continuous mixing in the following order: technological adjuvants (glycerol and sunflower oil), flavors, and spirulina powder (0%, 0.2%, 1% or 2% weight). After a final mixing (1 min), the resulting dough was spread in a stainless steel tray and cut into bars (40 ± 2 mm, width; 90 ± 3 mm, length; 12.5 ± 2 g, weight) by an extruder equipped with ultrasonic cutting technology (MIA Food Tech) (Figure 1, right). Bar basic color was evaluated on 6 bars per concentration, by visual observation, supported by the use of the Royal Horticultural Society Color Chart (RHSCC), sixth edition, placed in three different points free from dried fruit of each sample bar, to check the homogeneity of spirulina distribution. Finally, bars were promptly wrapped in single plastic packages to maintain their features during storage.

### 2.4 Sensory Evaluation

#### 2.4.1 Sample Preparation and Presentation

Packed dried fruit bars were labeled with three‐digit random numbers. Immediately before the test, the wrap was removed, and bars were served on a tray at room temperature (20°C ± 2°C). Sensory analysis was performed in individual booths equipped with tablets running FIZZ, specific software for sensory data acquisition (Biosystemès, Couternon, France), according to the standard protocol UNI ISO 8589:1990 (UNI Ente Italiano Di Normazione 1990), at the CNR sensory lab in Bologna.

#### 2.4.2 Panel Test Design and Execution

The sensory evaluation protocol included: DA, CATA, and TDS. Nine expert assessors with more than 70 h of training in sensory analysis, especially on energy bars, DA, CATA, and TDS, performed the sensory analysis. Data collection was performed by using the Fizz software. Before text execution, panelists were informed of the main research outcomes and gave consent for their data to be used. Participation in the research was voluntary, and the right to privacy and data protection was respected based on current legislation (GDPR 2016/679).

##### 2.4.2.1 DA Test

Six sensory experts, from the IBE sensory team, compiled the terminology and rearranged the terms used in the literature (Onacik‐Gur et al. 2018; Bower and Whitten 2000; Zen et al. 2020) or added news terms to create an appropriate lexicon list for the DA. Thirty‐two sensory attributes were used for the evaluation. Nine olfactory and aromatic attributes were chosen: dried fruit, red fruits, honey, raisins, citrus, peanut, toasted, spicy and off‐odor/off‐flavor. Moreover, five gustatory and eight texture attributes were also evaluated: sweet, acid, bitter, salty, and umami; and firmness, crunchiness, adhesiveness, gumminess, graininess, moisture, pungency, and astringency. DA tests, performed by the expert panel, were carried out in duplicate, and samples were presented monadically in a balanced order. The descriptors were rated on a 9‐point scale from “1: no perception” to the “9: highest intensity perceivable”. The panelists were also asked to rate the products' overall liking on a 9‐point hedonic scale from 1: extremely disliked; “5: neither liked nor disliked”; to “9: extremely liked”. Panelists used water to rinse their mouths between samples.

##### 2.4.2.2 CATA Test

A focus group was performed to elicit sensory attributes of spirulina. The panelists evaluated 50 mL 1% spirulina water solution and elicited olfactory and flavor attributes they perceived. The most cited olfactory and aromatic attributes, in line with the literature (Kuatrakul et al. 2017), were chosen to perform a CATA test, soon after the DA test, to highlight any possible influence of spirulina perception on dried fruit bar. Eight aromatic and olfactory attributes were chosen for the CATA test: mushroom, vegetables, cooked vegetables, herbaceous, seaweed, legumes, soy, and no sensation. Then, panelists were asked to check among the list of aromatic and olfactory attributes the sensation that described the product's sensory perception the most (Ares et al. 2010).

##### 2.4.2.3 TDS Test

Nine expert panelists were instructed to check, among a list of attributes, the one catching their attention, indicated as dominant, for 60 s, the whole duration of the tasting, aftertaste included (Wagner et al. 2023). The attributes list, selected from the literature (Onacik‐Gur et al. 2018; Bower and Whitten 2000) was as follows: dried fruit, red fruits, honey, raisins, citrus, peanut, toasted, spicy, and seaweed flavor. Attribute order was randomized among participants to reduce potential bias due to the attribute position. Samples (8 g) were served in organic plastic cups, labeled with three‐digit random numbers, on a tray at room temperature (20°C + 2°C). The test starts when the assessors put the whole sample in their mouths and immediately click start. When the dominant perception changed, the panelists indicated the new dominant sensation until the perception ended. Participants were free to choose the same attribute several times or never select an attribute.

### 2.5 Statistical Analysis

Data were analyzed using IBM SPSS V. 27; R programming language ver. 4.3.1 (R Core Team 2023. _R: A Language and Environment for Statistical Computing. R Foundation for Statistical Computing, Vienna, Austria) and SensoMineR: Sensory Data Analysis for R package version 1.2. Shapiro–Wilk's test was performed on data from descriptive analysis to make sure that the data are from a normal distribution. In the situations where the assumptions are violated, non‐paramatric tests are performed. In this case, a Kruskal–Wallis test by rank (a non‐parametric alternative to a one‐way ANOVA test, which extends the two‐sample Wilcoxon test in the situation where there are more than two groups) was performed. The significance level was fixed at *p* < 0.05. Mean DA intensity values were used to generate a spider plot and to represent the bar sensory profiles. A principal component analysis (PCA) was performed on the matrix of the average scores of each sensory attribute to summarize through a map the main bars' characteristics. A contingency table was obtained by CATA responses, and Cochran's Q‐test was performed to analyze cross‐tabulation data.

## 3 Results and Discussion

### 3.1 Industrial Manufacturing of an Innovative Cereal Bar

**FIGURE 2:**
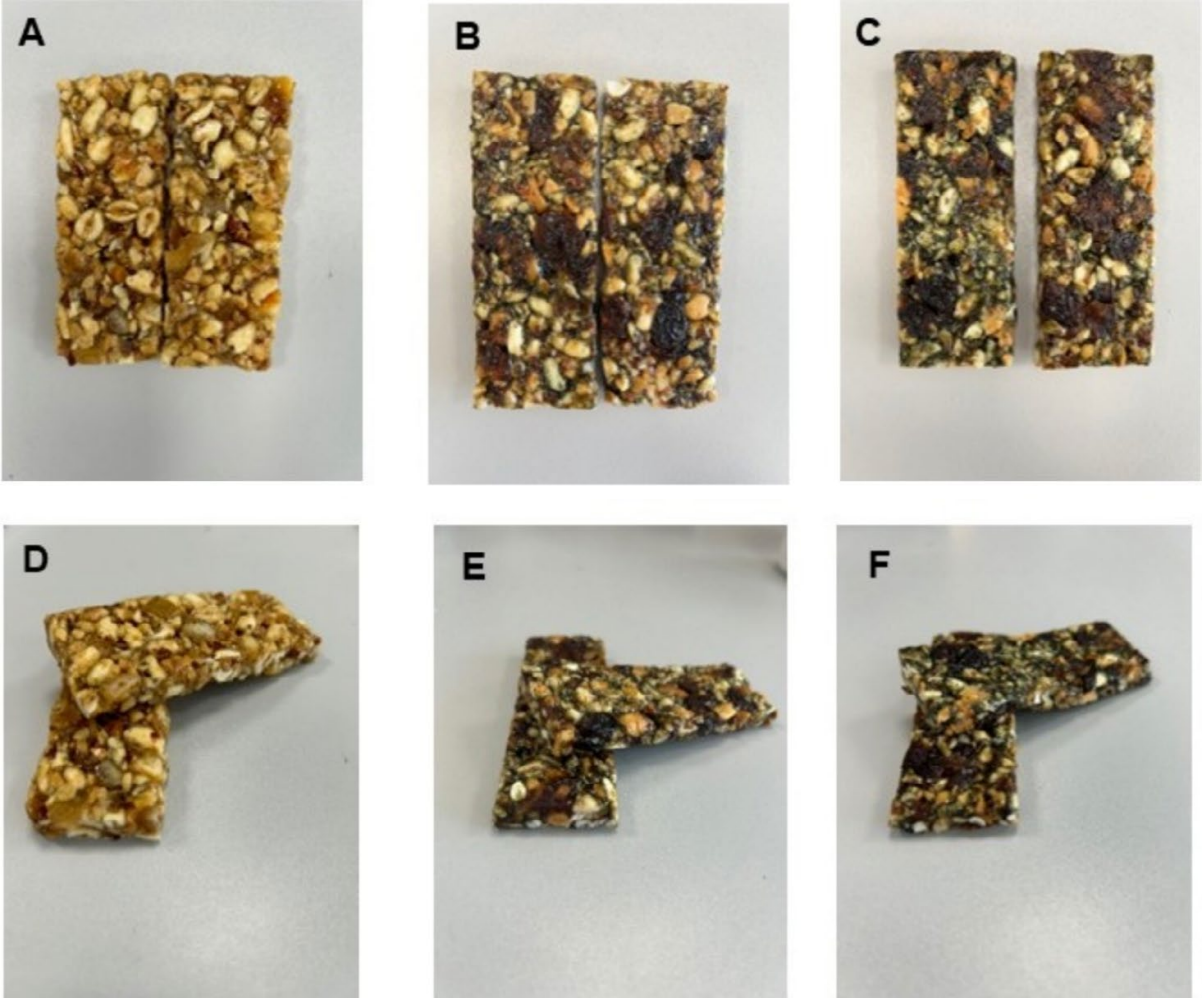
Appearance of packed bars with different percentages of Spirulina powder (0%–2%). (A, D) 0.2%; (B, E) 1%; (C, F) 2%.

The procedure adopted for producing cereal bars supplemented with candied and dried fruits led to the production of suitable snack products. Spirulina had an evident impact on bar appearance (Figure 2). Color indicated a homogeneous spirulina distribution in dough, despite the heterogeneous placement of the solid fruit components (Figure 2), in line with other similar cereal bars on the market. A recent study has reported the formulation of cereal bars enriched with *Pereskia aculeata* Miller powder, providing interesting bioactive molecules and nutrients (da Cruz et al. 2024). This is evidence of the broad potential of innovation in cereal bar ingredients and product development.

### 3.2 Nutritional and Functional Parameters

Nutritional data (Table 2A), derived from the US Department of Agriculture nutritional tables, have been elaborated to define the main nutritional parameters of the experimental bars (Table 1) and indicate a protein content increase correlated to spirulina concentration (0.2%: +1% proteins; 1.0%: +5.6% proteins; 2.0%: +10.7% proteins). According to spirulina composition, C‐phycocyanin was the major added protein in bars, rich in antioxidant properties (Hussein et al. 2015), and ranging between 46.65 and 54.65 mg/g spirulina biomass (Khandual et al. 2021). As shown in Table 2A, the addition of spirulina induces very slight changes in fat, carbohydrate, fiber, polyphenols, and minerals concentration, except iron (2.0%: +21%), potassium (+10%), copper (+7%), calcium (+6%), and sodium, increasing from 1 mg concentration in the control bar to 22 mg in the 2.0% spirulina‐enriched bar. However, apart from sodium, these changes have a negligible impact on the overall nutritional bar's profile considering the size of the commercial portion (30 g). Moisture, calculated by LoD, and the estimated calories remained stable in the various samples. The composition in a single serving (30 g) is reported in Table 2B. We also reported for 2% spirulina bars the percent daily value (% DV) of macro‐ and micronutrients, according to the daily value on the Nutrition and Supplement Facts Labels by US Food and Drug Administration (https://www.fda.gov/food/nutrition‐facts‐label/daily‐value‐nutrition‐and‐supplement‐facts‐labels). The reported % DV highlighted the nutritional contribution in minerals of the functional bars.

**TABLE 2A:** Nutritional profile of control (0) and spirulina‐enriched (0.2%; 1%; 2%) cereal bars.

100 g bar	Spirulina content (%)			
	0	0.2	1.0	2.0
Macronutrients				
Moisture (%)	6.96	6.96	6.90	6.85
Protein (mg)	10.74	10.86	11.32	11.89
Fat (mg)	20.21	20.23	20.29	20.37
Carbohydrate (mg)	11.90	11.94	12.13	12.37
Fiber (mg)	10.40	10.40	10.43	10.47
Energy (Kcal)	460.76	461.34	463.66	466.56
Minerals				
Ca (mg)	39.58	39.82	40.78	41.98
Fe (mg)	2.71	2.76	2.99	3.28
Mg (mg)	78.69	79.08	80.64	82.59
P (mg)	164.88	165.12	166.06	167.24
K (mg)	250.05	252.77	263.65	277.25
Na (mg)	1.06	3.16	11.56	22.06
Zn (mg)	1.13	1.13	1.15	1.17
Cu (mg)	0.23	0.24	0.29	0.35
Mn (mg)	0.74	0.74	0.76	0.78
Se (mg)	11.85	11.87	11.92	12.00
Poliphenols (mg GAE)	58.85	58.91	59.14	59.43
Moisture^a^ (%)	6.96	6.96	6.90	6.85

^a^ Data were derived from USDA FoodData Central (U.S. Department of Agriculture, Agricultural Research Service 2024), excluding moisture (a), which was measured using the LoD method.

**TABLE 2B:** Nutritional profile of control (0) and spirulina enriched (0.2%, 1%, and 2%) cereal bars in a single serving (30 g) and recommended daily percentage of macro‐ and micronutrients.

30 g bar	Spirulina content (%)				% DV*
	0	0.2	1.0	2.0	
Macronutrients					
Moisture^a^ (%)	2.09	2.09	2.07	2.06	
Protein (mg)	3.22	3.26	3.40	3.57	7.13
Fat (mg)	6.06	6.07	6.09	6.11	7.83
Carbohydrate (mg)	3.57	3.58	3.64	3.71	1.35
Fiber (mg)	3.12	3.12	3.13	3.14	11.22
Energy (Kcal)	138.23	138.40	139.10	139.97	
Minerals					
Ca (mg)	11.87	11.95	12.23	12.59	1.26
Fe (mg)	0.81	0.83	0.90	0.98	6.56
Mg (mg)	23.61	23.72	24.19	24.78	7.08
P (mg)	49.46	49.54	49.82	50.17	5.02
K (mg)	75.02	75.83	79.10	83.18	2.38
Na (mg)	0.32	0.95	3.47	6.62	0.28
Zn (mg)	0.34	0.34	0.35	0.35	2.34
Cu (mg)	0.07	0.07	0.09	0.11	5.25
Mn (mg)	0.22	0.22	0.23	0.23	4.68
Se (mg)	3.56	3.56	3.58	3.60	100.00

* %DV in bars containing 2% spirulina.

Functional parameters were explored through reported food applications of different spirulina concentrations in various foodstuffs and matched with our cereal bars' ideal features. The spirulina powder maximum concentrations (2%), resulting adequate for industrial production, were selected based on previous data on snacks (Lucas et al. 2018) and other foodstuffs (Table 3). Data reported in Table 3 underlined that spirulina content largely varied among different food applications. As shown in other studies related to different food types, 3% *A. platensis* incorporation was necessary to exert anti‐oxidant properties in gluten‐free pasta (Fradinho et al. 2020). The combination of at least 6% spirulina and sourdough technology was required to develop bakery products with functional properties (Niccolai et al. 2019). The tested spirulina ratio was even higher in durum‐wheat pasta (Raczyk et al. 2022). In the application of spirulina powder in other matrices, like dairy products (Barkallah et al. 2017; Patel et al. 2019) or meat (Marti‐Quijal et al. 2019; Luo et al. 2018), the percentage of applied spirulina largely varied based on the type of employed foodstuff and needs for a specific health‐enhancing effect.

**TABLE 3:** Main current food applications of spirulina.

Food	Spirulina %	Health effect	References
Sourdough crostini	2.0–10.0	Higher antioxidant capacity	Niccolai et al. (2019)^a^
Cookie doughs	0.5–2.0	Greater accessibility of P, K, Ca, Mg, Fe, Zn, and Se content	Uribe‐Wandurraga et al. (2020)^b^
Durum wheat pasta	3.0–10.0	Improved amino acid profile and higher total fiber content	Raczyk et al. (2022)
Gluten‐free pasta	1.0–3.0	Higher antioxidant activity and digestibility	Fradinho et al. (2020)
Cereal snacks	2.6	Overall increase of nutrients	Lucas et al. (2018)
Yogurt	0.25–1.0	Improved antioxidant activity	Barkallah et al. (2017)
Yogurt	7.0	Increase in probiotic viability and carotenoid content	Patel et al. (2019)
Meat (sausages and burgers)	1.0–5.0	Inhibition of lipid oxidation	Marti‐Quijal et al. (2019); Luo et al. (2018)

^a^ According to the European Commission Regulation on nutritional claims, “crostini” incorporated with 6% and 10% biomass can be claimed to be a “source of protein”.

^b^ Cookies enriched with 1.5 or 2% of Chlorella or Spirulina are foods classed as “high in selenium”.

The three spirulina concentrations chosen for this study fit industrial production requirements and comparative sensory analyses. The highest concentration tested was the same, identified by Souiy et al. (2022) as the best formulation in terms of nutritional, physical, microbial, and sensory characteristics in cereal bars enriched with spirulina and flavored with neroli. Importantly, 2% spirulina provided substantial functional properties to the bars based on the specific presence of C‐phycocyanin with known anti‐inflammatory and anti‐oxidant effects (Liu et al. 2022).

### 3.3 Sensory Analysis

#### 3.3.1 DA Profile Results

**FIGURE 3:**
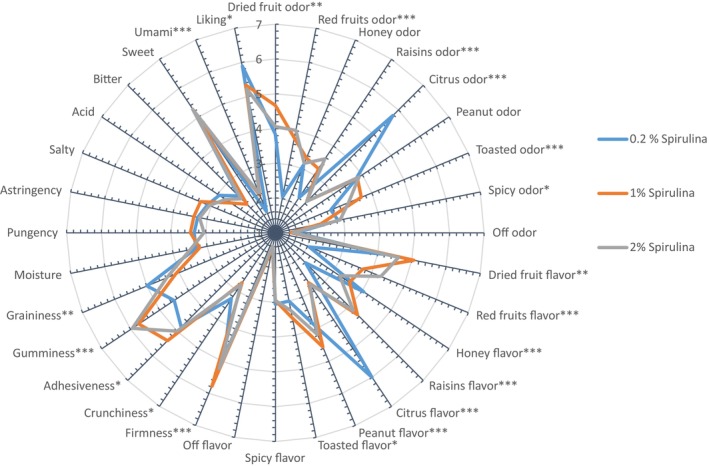
DA profiles of the dried fruit bars.

ANOVA performed on panel sensory data highlighted clear sensory differences among the dried fruit bars with low and high spirulina concentrations (Table 4 and Figure 3). Statistical differences were mainly related to olfactory and aromatic traits, together with mouthfeel sensations. The 0.2% spirulina bar was characterized by citrus olfactory and aromatic notes and by honey flavor. The 1% spirulina bar was described by dried and red fruits, raisins, and toasted olfactory notes. In the highest spirulina concentration bar, 2%, the olfactory and aromatic notes were mainly red fruits and raisins, together with a toasted and spicy odor. Regarding the mouthfeel sensations, the 0.2% spirulina bar was higher in graininess, while the 1% and 2% spirulina bars were characterized by high gumminess (5.9 in the 2% bar and 5.7 in the 1% bar) and firmness, especially in the 1% bar (5.8).

**TABLE 4:** Mean intensity scores for the sensory attributes of the different dried fruit bar.

Spirulina percentage	Firmness*	Crunchiness	Adhesiveness	Gumminess***	Graininess*	Moisture	Pungency	Astringency	Salty	Acid	Bitter	Sweet	Umami	Liking
0.2	4.8 b	3.3	4.8	4.5 b	5.0 a	3.3	3.3	3.3	3.1	2.9	2.5	5.2	1.7	5.9
1%	5.8 a	2.7	5.4	5.7 a	4.1 b	3.2	3.4	3.4	3.3	2.6	2.2	5.1	2.2	5.3
2%	5.2 b	2.8	5.0	5.9 a	4.4 b	3.5	3.1	3.2	3.1	2.7	2.4	5.3	2.2	5.2

Spirulina percentage	Odors									Flavors								
	Dried fruit *	Red fruits ***	Honey	Raisins**	Citrus***	Peanut	Toasted**	Spicy*	Off odor	Dried fruit*	Red fruits ***	Honey*	Raisins***	Citrus***	Peanut*	Toasted	Spicy	Off flavor
0.2	3.8 b	2.1 b	3.1	2.2 b	5.8 a	3.8	2.7 b	2.8 ab	1.6	4.2 b	2.1 b	4.1 a	2.2 b	6.0 a	3.8 b	3.0	3.1	1.4
1%	4.7 a	3.8 a	3.3	3.2 a	2.3 b	3.8	3.7 a	2.3 b	1.4	5.1 a	3.7 a	3.6 b	4.3 a	2.7 b	4.6 a	3.5	2.9	1.4
2%	4.1 b	4.0 a	3.2	3.6 a	2.3 b	3.9	3.3 a	2.9 a	1.5	4.6 ab	4.3 a	3.2 b	4.2 a	2.8 b	4.2 b	3.3	2.9	1.4

*Note:* Different letters correspond to different means according to the Kruskal–Wallis test.

* *p* < 0.05.

** *p* < 0.01.

*** *p* < 0.001.

#### 3.3.2 PCA Profile Results

**FIGURE 4:**
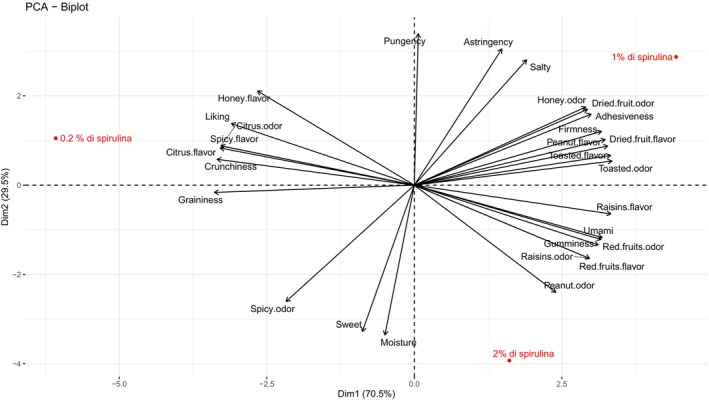
Representation of the samples and attributes in the first two dimensions of the PCA analysis using DA data.

DA results, obtained through panel test, were also analyzed in a multidimensional way through PCA, which showed that the first and the second principal components accounted for 70.5% and 29.5%, respectively, of the experimental variability of the data, representing 100% (Figure 4). The three samples were clearly separated into three quarters and characterized by different attributes. The first dimension (70.5% of variance), representing most of the variability, was positively correlated with red fruits and dried fruit notes, raisins, and mouthfeel sensations (firmness, adhesiveness and gumminess), that mainly characterized 1% and 2% bars, but also with peanut and toasted odor and flavor. Moreover, the first dimension was negatively correlated with citrus odor/flavor, spicy flavor, and honey flavor, crunchiness, and graininess attributes that described the 0.2% bar, while showing a clear correlation with the overall liking vector. The second dimension (29.5% of variance) was positively correlated with the attributes: pungency, astringency, and salty, mainly related to the 1% bar features, and negatively correlated with spicy odor, sweet, and moisture, mainly describing the 2% bar.

#### 3.3.3 CATA Results

**FIGURE 5:**
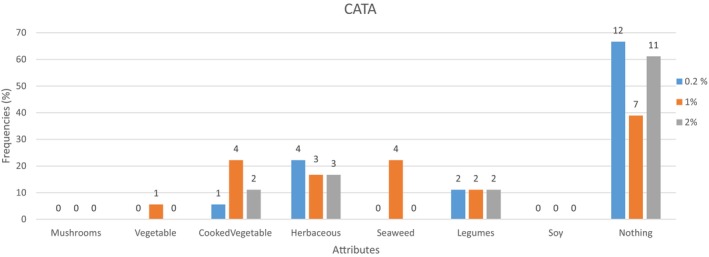
Frequency count of the aromatic attribute by sample.

Eighteen responses were collected through CATA analysis. The citation frequencies were low for most of the attributes, meaning that the panel did not use them to describe the samples (Figure 5 and Table 5). The 1% spirulina bar was the only one showing a slight spirulina aromatic influence on the flavor composition; indeed, the overall seaweed perception (Table 5) in the 1% bar was higher (14 citations) than in the other two bars (7 citations each). The only significant difference was related to the “seaweed” attribute, meaning that this attribute fits the 1% bar's description.

**TABLE 5:** Frequency count of the aromatic attribute by sample.

Product	Chosen attributes						Overall spirulina's notes perception
	Vegetable	Cooked vegetable	Herbaceous	Seaweed	Legumes	Nothing	
0.2% spirulina	0	1	4	0	2	12	7
1% spirulina	1	4	3	4^a^	2	7	14
2% spirulina	0	2	3	0	2	11	7

^a^ The green‐labeled cell indicates a statistically significant difference according to Cochran's Q‐test (*p* < 0.05).

#### 3.3.4 TDS Results

**FIGURE 6:**
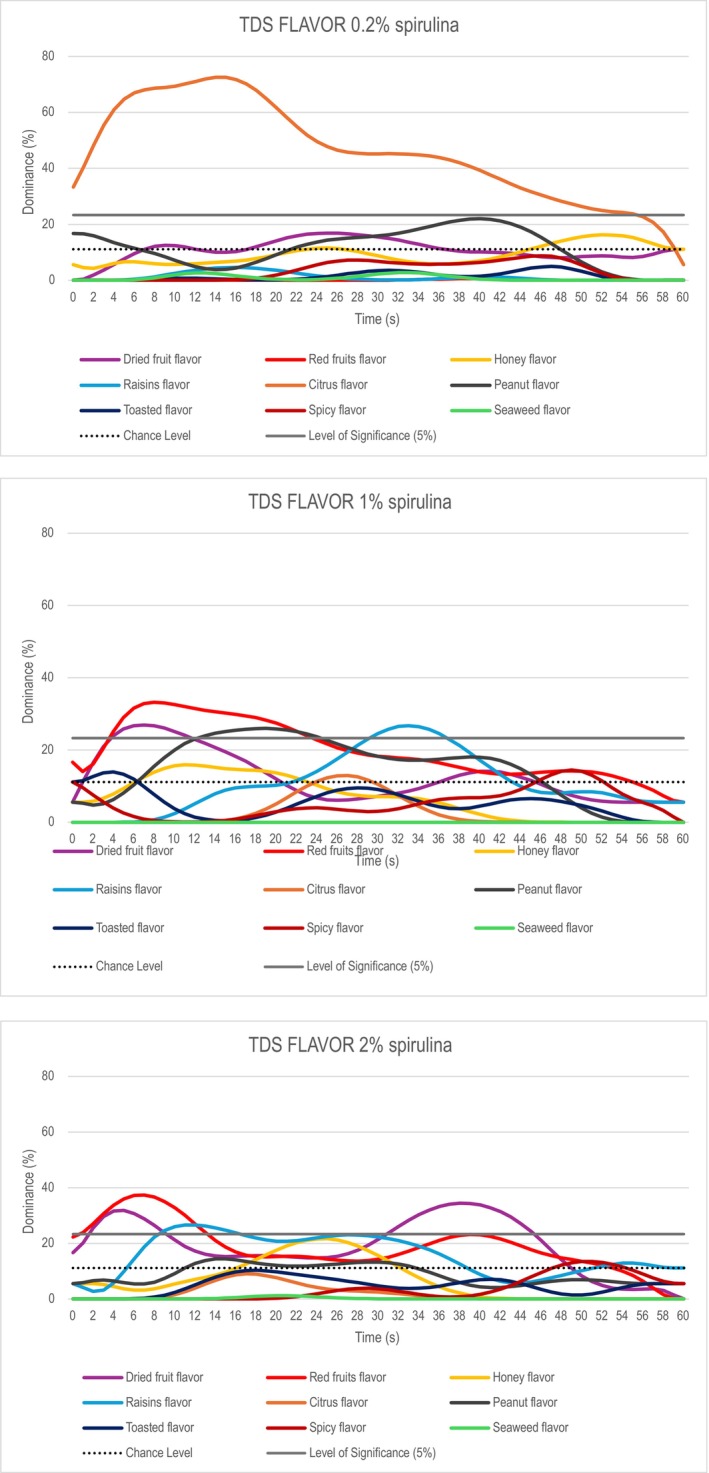
TDS Graphical representation for 0.2% (upper panel), 1% (middle panel), and 2% spirulina bar (bottom panel).

TDS graphs for the aromatic attributes of the three Spirulina bars are represented in Figure 6. The dominance duration was the same for all the three samples and covered the whole taste duration (60s). In the 0.2% bar sample, the attribute “citrus” was dominant throughout the taste duration covering other attribute perceptions. Therefore, the “peanut” flavor registered a dominance rate higher than the chance level in the first 4 s and between 22 and 48 s. “Dried fruit” flavor and “honey” had also a dominance rate higher than the chance level respectively between 4 and 40 s and between 42 and 58 s. The 1% and 2% bars showed a different trend with the dominance of “red” and “dried fruit” flavors. In the 1% bar, the “red fruit flavor” was dominant for the first 22 s together with “dried fruit” (12 s) and “peanut flavor” (12‐22s), then the “raisins flavor” appeared between 28 and 38 s. Moreover, the “red fruits” flavor registered dominance rate greater than the chance level for the whole dominance duration. In the 2% bar “red fruits” was dominant for the first 14 s together with “dried fruit” (8 s) and raisins (10–16 s). Then the “dried fruit” flavor became the only dominant flavor between 30 and 46 s. The “raisins” flavor had a dominance rate that was greater than the chance level between 4 and 40 s, “honey” flavor as well as “red fruits” also registered a dominance rate greater than the chance level respectively between 18 and 30 s and 30 and 52 s.

#### 3.3.5 Sensory Results Discussion

The integrated and comprehensive sensory approach used, involving the traditional method (DA), a dynamic test (TDS) and a rapid innovative method (CATA), applied to highlight any adverse aromatic note, gave a complete overview of the cereal bars' sensory features. As compared to other studies about sensory evaluation of bars enriched with spirulina (Fanari et al. 2023), our approach provides a wider range of descriptors, offering a more detailed profile. Furthermore, this is the first report on the application of TDS, to this product type. In other studies related to cereal bars enriched with *Pereskia aculeata* Miller, powder, a sensory profile was not determined, but only a consumer hedonic evaluation was performed (da Cruz et al. 2024). The sensory analysis tests showed that spirulina addition in bars did not elicit any negative effect on the sensory characteristics, in agreement with Hussein et al. (2015) and Lucas et al. (2018). Spirulina addition, at higher concentrations (1% and 2%), had a significant impact on the flavor composition; however, the combination with the other ingredients used resulted in pleasant red fruits, raisin, and dried fruits notes. No negative effect was evidenced by spirulina presence; only a slight seaweed note was highlighted, on the bar with 1% of spirulina. This is in line with Spínola et al. (2024) who showed that at lower concentrations (1%), the seaweed flavor of spirulina might be more noticeable because it is not masked by other ingredients. Furthermore, it highlighted that spirulina's complex chemical composition, including its high levels of protein, essential fatty acids, vitamins, minerals, and bioactive compounds like phycocyanin can contribute to various flavor profiles. These results showed that the manufactured products have promising sensory characteristics since they can mask spirulina taste and smell potentially affecting sensory acceptance (Onacik‐Gur et al. 2018). Importantly, these findings represented a major difference from previously published reports in which the presence of similar or lower percentages of spirulina powder dramatically influenced consumer acceptance (Lucas et al. 2018; Fradinho et al. 2020). Furthermore, our study highlighted a cheap alternative to the use of micro‐encapsulated spirulina (Zen et al. 2020) or coaxial printing methodology (Uribe‐Wandurraga et al. 2020) for food applications, which is the combination with selected ingredients. Texture also plays an important role in sensory acceptance (Bower and Whitten 2000). Our analysis showed a texture change in spirulina‐enriched bars as they got gummy and firm as the spirulina concentration increased. These observations are in line with El Nakib et al. (2019), who showed the advantage of spirulina addition that helps maintain integrity and reduces breakage during packaging and distribution. Moreover, snack enrichment with 2.6% spirulina powder has been shown to improve most nutritional parameters with a sensory acceptance index of 82% (Lucas et al. 2018).

## 4 Conclusions

Cereal dried fruit bars, chosen for this research, are considered a promising functional food, thanks to their bio‐nutritive components, which make them a meal substitute and a fast source of energy and nutrients that can be further improved with bioactive compound addition. Spirulina addition, studied in our research, increases bars' nutritional levels as well as protein content. This study proved that the innovative cereal‐dried fruit bar formula can benefit from spirulina powder addition, providing high‐value antioxidant protein, while maintaining optimal organoleptic features. Indeed, sensory analysis showed that bar odor and flavor were not influenced by any spirulina's unpleasant organoleptic note. Future work will be addressed to explore the spirulina cereal bars acceptance on a larger and distinctive consumer panel by applying the herein‐identified sensorial parameters. The manufactured bars are a nutritious snack that could represent a good instrument to promote healthy diets, providing ready‐to‐eat food and improving consumer lifestyle and dietary intake of bioactive compounds.

## Author Contributions

**Antonio Ottombrino:** investigation (equal), methodology (equal). **Marta Cianciabella:** investigation (equal), software (equal). **Chiara Medoro:** data curation (equal), investigation (equal), software (equal), validation (equal), writing – original draft (equal), writing – review and editing (equal). **Adelia Picariello:** investigation (equal). **Annachiara Oliviero:** investigation (equal), methodology (equal), writing – original draft (equal). **Vincenzo De Sena:** validation (equal). **Stefano Predieri:** conceptualization (equal), formal analysis (equal), funding acquisition (equal), validation (equal), writing – original draft (equal), writing – review and editing (equal). **Mauro Rossi:** conceptualization (equal), funding acquisition (equal), writing – original draft (equal), writing – review and editing (equal).

## Ethics Statement

The sensory experiment was conducted in agreement with the Declaration of Helsinki. No ethics approval was required, since this study involved a panel of experienced and trained assessors, who were regularly involved in sensory evaluation of food products.

## Consent

Written informed consent was obtained from all study participants.

## Conflicts of Interest

The authors declare no conflicts of interest.

## Data Availability

Data supporting reported results are available from the corresponding author [C.M.] upon reasonable request.
